# Lectures upon the Principles of Surgery

**Published:** 1900-11

**Authors:** 


Lectures Upon the Principles of Surgery. Delivered at the University of Michigan. By Chas. B. Nancrede, A. M., M. D., LL. D., Professor of Surgery and of Clinical Surgery Philadelphia Polyclinic, Etc. With an appendix containing a resumé of the principal views held concerning inflammation, by Wm. A. Spitzley, A. B., M. D., Senior Assistant in Surgery, University of Michigan. Illustrated. Pages, 398; price, $2.50. Philadelphia: W. B. Saunders, 925 Walnut Street. 1899.

This work, as the title implies, is devoted to a discussion of the principles of surgery. It has little to do with operative surgery, and is intended mainly for teachers and for students. These lectures are valuable. The theories are ably discussed, and especially that pertaining to inflammation. The appendix, giving a resumé of the principal views on inflammation, is particularly interesting.

